# Insights on pediatric medication initiation: perceptions of caregivers and children

**DOI:** 10.3389/fphar.2025.1612169

**Published:** 2025-06-06

**Authors:** Maria Rubio-Valera, Cristina Carbonell-Duacastella, Maite Peñarrubia-María, Eva Pacheco, Patricia Gabriela-Ricciardeli, Ignacio Aznar-Lou, Montserrat Gil-Girbau

**Affiliations:** ^1^ Health Technology Assessment in Primary Care and Mental Health (PRISMA) Research Group, Teaching, Research and Innovation Unit, Institut de Recerca Sant Joan de Déu, de Llobregat, Spain; ^2^ Parc Sanitari Sant Joan de Déu, Sant Boi de Llobregat, Spain; ^3^ CIBER of Epidemiology and Public Health (CIBERESP), Madrid, Spain; ^4^ School of Medicine and Health Sciences, Universitat de Barcelona, Barcelona, Spain; ^5^ Primary Care Center Bartomeu Fabrés Anglada, Gerencia Territorial Metropolitana Sud, Institut Català de la Salut, Gavà, Spain; ^6^ Fundació Institut Universitari per a la recerca a l’Atenció Primària de Salut Jordi Gol i Gurina (IDIAPJGol), Barcelona, Spain; ^7^ Primary Care Center Alhambra, Hospitalet de Llobregat, Direcció d’Atenció Primària Costa de Ponent, Institut Català de la Salut, Barcelona, Spain; ^8^ Research Network on Chronicity, Primary Healthcare and Health Promotion (RICAPPS), Barcelona, Spain

**Keywords:** pediatrics, medication adherence, qualitative research, medication, patient participation

## Abstract

**Introduction:**

Children fail to initiate a high proportion of medications but little is known about motivations for non-initiation.

**Objective:**

To explore the factors affecting the decision to initiate a medication prescribed to children from caregivers’ and children’s perspectives.

**Methods:**

Qualitative study based on Grounded theory using a constructivist approach. Twenty-one caregivers and six children (<18 years old) were individually interviewed between 2021 and 2022 in Spain using a semi-structured thematic guide. Interviews were transcribed and analyzed by pharmacists, pediatricians and family physicians through a constant comparative analysis and results were internally audited.

**Results:**

Caregivers and healthcare professionals are the central figures involved in the decision-making process regarding treatment initiation; children were rarely involved. Caregivers, usually mothers, made a risk-benefit evaluation based on the perception of the disease and the medication, which was influenced by intrapersonal factors (emotional burden, health literacy and stigma); children-related factors (age, treatment and emotional burden); and factors related to the professionals (accessibility, discourse alignment, information, respect and emotional support, trust and specialty); healthcare system (trust and use of e-consultations) and context (media, peer pressure and social stigma).

**Conclusion:**

The decision to initiate medication in the pediatric population is multifactorial and influenced by perceptions on the disease and treatment, intrapersonal factors related to the caregivers and children, and interpersonal factors and factors related to the healthcare system and contexts. An informed, shared decision-making process that considers both the participation of children and the needs for support from caregivers when prescribing a treatment could promote initiation in the pediatric population.

## 1 Introduction

Optimal use of medicines is vital for preventing and managing illnesses. Medication non-adherence results in poorer health outcomes and higher economic burden on the healthcare system (Aznar-Lou et al., 2017; McGrady and Hommel, 2013). Between 9% and 32% of medications prescribed to children are not initiated (Carbonell-Duacastella et al., 2022; Rosman et al., 2012), compounding the impact of early discontinuation and poor implementation.

Most frameworks for understanding health behaviors, including adherence, are adult-centered (Holtzman et al., 2015; Fisher et al., 2003; Gil-Girbau et al., 2020; Peñarrubia-María et al., 2022), although some have been adapted to children and/or adolescents (hereafter referred to as “children”) (Dima et al., 2013; Jaccard et al., 2002; Guilamo-Ramos et al., 2008). A specific model for the pediatric population (De Civita and Dobkin, 2004) defines pediatric adherence as the manifestation of multiple behaviors related to the prescribed treatment, influenced by contextual and developmental characteristics, shaped by the disease, and interpreted by both the caregiver and the child. Pediatric adherence is therefore considered multidimensional and dynamic, involving mutually influential exchanges within and between three subsystems (the triadic association): caregiver-medical team, child-medical team, and caregiver-child. These interactions are affected by the disease course, contextual features, and the child’s developmental and adaptive capacities.

Quantitative studies show that non-initiation is influenced by factors related to the medication, the child, and the provider. It is more likely in acute treatments, in toddlers and adolescents, and when the prescription is issued by a family physician (FP) (Carbonell-Duacastella et al., 2022). However, quantitative studies alone are insufficient to explain a multifactorial behavior such as initiation. According to a review, parents’ and caregivers’ concerns about the treatment and condition typically affect adherence (Santer et al., 2014). Other factors, including treatment complexity, child resistance to treatment, the treatment’s interference with family relationships, and the desire to ensure a ‘normal life’ for the child, vary across conditions. However, the review focused on long-term adherence in chronic conditions. Factors influencing initiation may differ from those affecting treatment continuation and implementation. Non-initiation is also significant in acute pediatric treatments; for example, 3% of antibiotic prescriptions are not initiated (Carbonell-Duacastella et al., 2022).

One qualitative study examining non-initiation of acute and chronic treatments for mental and physical conditions in adults found that, regardless of disease type or duration, initiation is influenced by patients’ beliefs about medication and illness, emotional responses, health literacy, cultural factors, and their relationship with the healthcare system (Gil-Girbau et al., 2020; Peñarrubia-María et al., 2022). The few qualitative studies that have assessed barriers to initiation in pediatric treatments have focused on specific conditions–such as HIV, tuberculosis and attention/deficit disorder–and were conducted mainly in developing countries (Ahmed et al., 2017; Coit et al., 2020; Coletti et al., 2012). While some identified factors, such as fear of side effects and stigma, may apply to the European context, most findings are context-specific. Moreover, the results of these studies are only partially applicable to other medications groups.

Given the relative lack of evidence on factors affecting medication initiation in the pediatric population, we aimed to explore the reasons, attitudes, and beliefs underlying the pediatric decision-making process from the perspectives of caregivers and children.

## 2 Material and methods

The paper follows the Standards for Reporting Qualitative Research recommendations (O’Brien et al., 2014; Dossett et al., 2021).

This study was approved by the Research Ethics Committees at Sant Joan de Déu and Idiap Jordi Gol. All participants, and their guardians for those under 18, provided informed consent. Written consent was obtained, except during periods of COVID-19 restrictions, when oral recorded consent was obtained after participants were mailed the information sheet and consent form. Adult participants received 20€ to cover time and travel expenses.

### 2.1 Design

**FIGURE 1:**
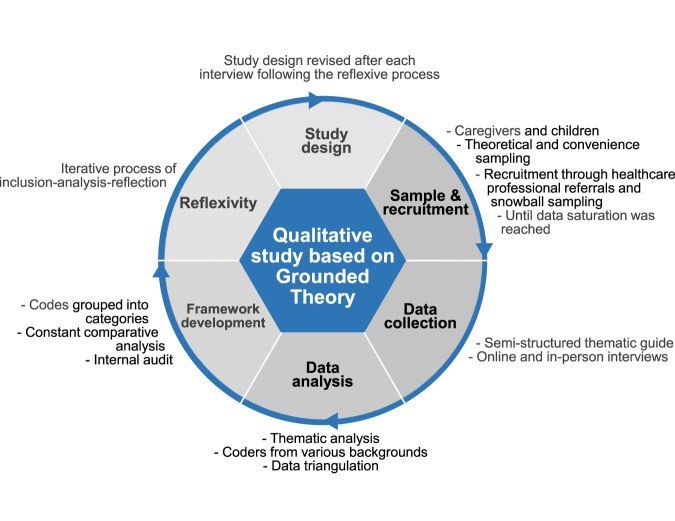
Summary of the research iterative methods.

Figure 1 illustrates the study methods. This was a qualitative study based on Grounded theory using a constructivist approach. Grounded theory was selected due to the lack of explanatory frameworks regarding the decision-making process for medication initiation in the pediatric population. The sampling-strategy and interview topics were informed by the Theoretical Model of medication non-initiation (Gil-Girbau et al., 2020; Peñarrubia-María et al., 2022) and the model developed by De Civita and Dobkin (2004). Following grounded theory principles, a constant comparative analysis was employed, and findings were compared with existing models (Fàbregues et al., 2006; Goulding, 2017).

The original study design and subsequent modifications–introduced after analysis and reflective discussions–were presented an external auditor group from the *Consorci de Salut i Social de Catalunya* before, during, and after the project. Feedback was integrated into the project where appropriate.

### 2.2 Context

The Catalan public health system offers universal healthcare to all residents (7.5 million) and is organized into health areas. Each citizen has a unique identification number, providing access to the entire public health system in Catalonia and Spain (Bernal et al., 2018).

Primary care (PC) provides first-level care for children and can refer patients to secondary care (SC). In PC, pediatricians care for individuals until age fifteen. After that, patients are generally seen by a FP, although family involvement in care is common. In SC, individuals are considered minors age eighteen.

Medicines covered by the public healthcare system are prescribed electronically and dispensed through community pharmacies. The level of payment contribution for children’s medicines depends on the type of illness (with reduced contribution for chronic conditions) as well as the designated parent’s (or legal guardian’s) income and employment status.

### 2.3 Research team

The research team comprised seven researchers (six female; five mothers), including FPs, pediatricians, and pharmacists with qualitative research expertise. Detailed researcher characteristics are available in Supplementary Material 1.

### 2.4 Sample and recruitment

Theoretical convenience sampling was used.

#### 2.4.1 Inclusion criteria

The sample included caregivers of children (≤18 years old) living in the province of Barcelona who had received at least one new prescription through the public health system and children who had received a new prescription and provided informed consent to participate. Initially, only caregivers of children who had not initiated a new treatment were included. However, due to difficulties in identifying such cases, the criteria were broadened after the initial interviews to include all children who had received a first prescription, regardless of treatment of initiation.

Heterogeneity criteria were applied based on disorder (acute/chronic; physical/mental), treatment (acute/chronic; route of administration), caregivers’ age and gender, children’s age and gender, nationality, socioeconomic status, and family structure.

Pediatricians, FPs, and community pharmacists recruited participants. Physicians and nurses identified eligible participants in PC, while community pharmacists identified them at the point of dispensing. A snowball sampling method was also used.

Professionals invited potential participants, explaining that a researcher would follow up to arrange the interview. During caregiver interview, we asked whether their child had participated in the decision-making process and if they would be willing to participate. One participant, an unaccompanied minor and ward of court, was contacted through legal guardians and interviewed after obtaining their consent.

Data collection continued until saturation was achieved. Saturation was considered to have been achieved when no new or relevant information emerged from interviews.

### 2.5 Data collection

Data collection occurred from February 2021 to February 2022 through an iterative process of inclusion, analysis, and reflection. Semi-structured interviews were conducted in Catalan and Spanish. Some interviews included both parents. Depending on participant preference and COVID-19 restrictions, interviews were held online of in person (in open spaces, at the participants’ home, or in our research center). Interviews averaged 43 min (range: 18–60 min) and were recorded.

Thirty-six families were identified; however, fifteen (42%) were excluded during recruitment (treatment had been initiated, participation declined, or contact was not possible). Nineteen caregiver interviews (18 mothers, 3 fathers) and six child interviews were conducted (Tables 1, 2 show participant characteristics). Diverse degrees of maturity were observed among the interviewed children; however, their discourses were generally aligned.

**TABLE 1:** Participant caregivers’ characteristics.

I#P#^a^	Gender	Age	Nationality	Education^b^	Employed	Civil status	Cohabitation	Private health insurance	Gender of C/A	Age of C/A	Disease(s)	Treatment(s)
I1P1	Fem.	47	Spanish	High	No	Married	S&C	No	Fem.	11	Migraine	Flunarizine (oral)
I2P2	Fem.	42	Spanish and Equatorial	Medium	Yes	Married	S&C	No	Fem.	7	Allergic cough	Cough syrup (oral)
I3P3	Fem.	51	Spanish	High	Yes	Married	S&C	No	Fem.	12	Acne	Benzoyl peroxide/Clindamycin (topical)
I4P4	Male	45	Spanish	High	Yes	Married	S&C	No	Male	13	Allergy	Cetirizine (oral)
I5P5	Fem.	33	Spanish	Medium	Yes	Married	S&C	Yes	Fem.	3	a) Constipation b) Infection	a) Osmotic laxative (oral) b) Antibiotic (oral)
I6P6 I6P7	Fem. Male	47 50	Spanish Spanish	High Medium	Yes No	Married Married	S&C	Yes Yes	1) Male 2) Fem.	1) 9 2) 6	1) Otitis 2) Bronchitis	1) Ciprofloxacin/Fluocinolone (otic); Amoxicillin/clavulanic acid (oral) 2) Prednisolone steaglate (oral)
I7P8	Fem.	33	Spanish and Dominican	Medium	Yes	Married	S&C	Yes	1) Fem. 2) Male	1) 1 2) 3	1a) Ear inflammation 1b) Conjunctivitis 2) Bronchitis	1a) Cortisone (topic) 1b) Neomycin/Polymyxin B Sulfate/Gramicidin (topic) 2) Montelukast (oral); Budesonide (inhaled)
I8P9	Fem.	49	Spanish	High	No	Married	S&C	Yes	Fem.	16	Acne	Isotretinoin (oral)
I9P10	Fem.	44	Spanish	Low	No	Married	S&C	No	Male	2	Epilepsy	Levetiracetam (oral)
I10P11	Fem.	57	Spanish	Low	Permanent disability	Single	Child	No	Male	16	Schizophrenia	Clozapine; lithium; valproic acid, aripiprazole, lorazepam (oral)
I11P12	Fem.	46	Spanish	High	Yes	Married	S&C	Yes	1) 1) Fem. 2) 2) Fem.	1) 7 2) 4	1) ADHD 2) Bronchitis/Pneumonia	1) Methylphenidate (oral) 2) Salbutamol; Budesonide (inhaled); Montelukast (oral)
I12P13 I12P14	Fem. Male	50 53	Spanish Spanish	High Medium	Yes Yes	Married Married	S&C	No No	Male	11	a) Allergy b) ADHD	a) Antihistamine (oral) b) Methylphenidate (oral)
I13P15	Fem.	58	Spanish	High	Yes	Married	S&C	Yes	Fem.	18	Acne	Isotretinoin (oral)
I14P16	Fem.	56	Spanish	High	Yes	Married	Partner, child and other relatives	Yes	Fem.	25*	Asthma	Montekukast (oral)
I15P17	Fem.	38	Spanish	Medium	Yes	Married	S&C	Yes	Male	7	a) Nocturnal enuresis b) Anxiety	a) Desmopressin (oral) b) Diazepam (oral)
I16P18	Fem.	49	Spanish	Low	Yes	Married	S&C	Yes	Fem.	12	a) Epilepsy b) Early Puberty	a) Levetiracetam (oral) b) Metformin (oral)
I17P19	Fem.	46	Spanish	High	Yes	Single	S&C	No	Male	11	Asthma and bronchitis	Budesonide (inhaled); Prednisolone steaglate (oral)
I18P20	Fem.	50	Spanish	Medium	Yes	Married	Spouse, child and spouses’ child	No	Fem.	14	Migraine and anxiety	Amitriptyline (oral)
I19P21	Fem.	39	French	High	Yes	Married	S&C	No	Fem.	6	Epilepsy	Levetiracetam; Topiramate; Stiripentol; Sodium valproate (oral)

ADHD: Attention-Deficit/Hyperactivity Disorder; C/A: Children/Adolescent; Fem.: female; S&C: spouse and children.

^a^ I#P#: number of interview and participant.

^b^ High: University degree; Medium: Secondary-level education and or Professional training, Low: compulsory education (until 16 years old).

*The patient was 6 years old when the new treatment for which the interview was conducted was prescribed.

**TABLE 2:** Participants’ characteristics: children.

Id	Gender	Age	Nationality	Education	Co-habitance	Private health insurance	Disease(s)	Treatment(s)	Caregivers interviewed (interview number)
C1	Fem.	12	Spanish	High school	1st degree family	No	Acne	Benzoyl peroxide/Clindamycin (topical)	Yes (I3)
C2	Fem.	16	Moroccan	High school	Guardians and other children*	No	a) Allergy b) Asthma	a) Cetirizine (oral) b) Budesonide (inhaled)	No
C3	Fem.	16	Spanish	High school	1st degree family	No	Allergy	Antihistamine (oral)	No
C4	Fem.	16	Spanish	High school	1st degree family	No	Gastroenteritis	Physiological serum; Paracetamol (oral)	No
C5	Fem.	16	Spanish	High school	1st degree family	Yes	Acne	Isotretinoin (oral)	Yes (I8)
C6	Male	11	Spanish	High school	1st degree family	No	a) Allergy b) ADHD	a) Antihistamine (oral) b) Methylphenidate (oral)	Yes (I12)

ADHD: Attention-Deficit/Hyperactivity Disorder; Fem.: female.

*The participant lived in an “Educative action residential center (CRAE)”, a shelter for children under government protection.

Interviews followed a semi-structured thematic guide (see the Supplementary Material) tailored for children and explored factors influencing the decision-making: health condition (type, severity, perceptions, and previous experiences), medication characteristics and experiences, prescription visit expectations and quality, relationships with healthcare professionals (prescriber and other professionals), family and caregiver influence, and social context (e.g., media and peer influence). Interview summaries were verified with participants at the end of each session.

Interviews were transcribed and pseudomized. Transcripts and audio files were securely stored at Parc Sanitari Sant Joan de Déu.

### 2.6 Analysis

Four researchers (CCG, MGG, PGR, and EP) with diverse backgrounds independently analyzed each interview using paper, boards, and ATLAS.ti. They extracted quotations, identified emerging themes, and assigned corresponding meanings and codes. The results were then triangulated and discussed until consensus was reached. The emerging codes were grouped into categories and progressively organized into a preliminary theoretical framework. MRV conducted an internal audit of the analysis, raising questions that were collaboratively addressed with the primary analysts to finalize the theoretical framework.

## 3 Results

**FIGURE 2:**
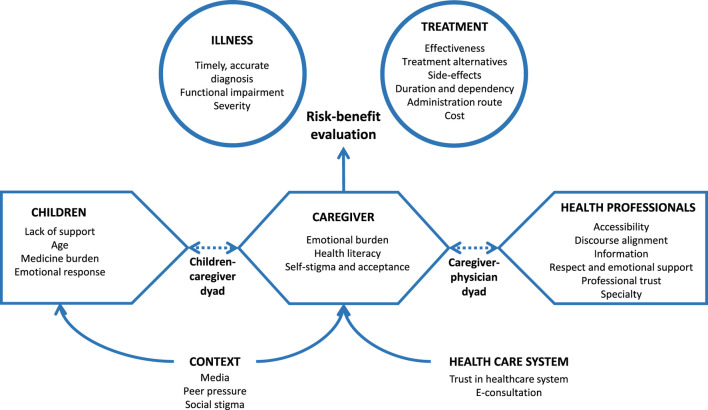
Theoretical model for pediatric medication initiation.

Figure 2 shows the theoretical model for pediatric medication initiation. After receiving a new prescription, caregivers (as decision-makers), children (as patients) and healthcare professionals (as providers) are the main decision-makers. To decide whether to initiate treatment, caregivers make a risk-benefit evaluation based on the perception of the disease and the medication, which is influenced by intrapersonal factors, child-related factors, and factors related to the professionals, healthcare system and context (which also affect children).

### 3.1 The children-caregiver and caregiver-physician dyads

Except for one participant in sheltered accommodation, the results indicate that mothers were the central figures who typically made the decision regarding children’s treatment. Mothers felt reassured when they shared the decision with spouses/partners, but paternal participation was rare.

*“No, the decision is mine. The decision was entirely mine. I told him [husband] and he said ‘Well, if it is okay with you, then go ahead’.” (I17P19)*

*“Not at all. My father and I never discuss anything. He has no idea about anything [her healthcare problems]. I do not know, my mother and I, I guess. She knows the most.” (C4)*

Despite purposive exploration, children maintained that they did not share decision-making with physicians. Furthermore, children were sometimes omitted from decision-making or indicated that, although their opinions were heard, caregivers had the final say.

*“No, no, look, the doctor called the center [where the child lives], the [social] educator answered the phone, that educator, who is not my guardian, and she answered the phone and she tells me, ‘[Name], you have to explain it to me so I can explain it to the doctor’. And I say fine, then that, and that, and she explains it to the doctor, and that’s it. And she tells me, ‘the doctor says that you have to buy a medicine’.” (C2)*

Notably, caregivers were more willing to accept the recommended treatment when physicians used the shared decision-making model and took a variety of other factors into consideration.

*“What also gives us lots of confidence is that the neurologist always suggests that the final say, that it is ours (…) She always told us that we are the ones who live with [Children], who know [Children], it is us (…) We reach a mutual agreement.” (I19P21)*

### 3.2 Illness

#### 3.2.1 Diagnosis accuracy and time-to-diagnosis delays

Sometimes caregivers and children were reluctant to accept treatment because diagnostic tests were not performed or because the cause of the problem was unknown (e.g., allergies with unknown triggers). Diagnosis and treatment recommendation delays were also associated with treatment rejection, while many families sought swifter diagnosis and treatment in private healthcare.

*“And we still do not know what this allergy is.” (C6)*

*“It was like, we do not know where it comes from [the health problem], so we do not know what to do [about the treatment] either (…) I think that she [children] should have started before [with the treatment] and most probably at this point, maybe she would not need it [the medication] anymore. But since it arrived late, of course [we decided not to use it].” (I12P14)*

#### 3.2.2 Severity and functional impairment

Caregivers and children sometimes preferred mild diseases to self-resolve. Children’s concerns about the disease positively influenced initiation. When the disease affected children’s functionality, both caregivers and children were more motivated to initiate.

*“At that time, I did not see my face [acne] that bad, you know? Now I do, but I did not before.” (C1).*

*“She [pediatrician] anticipated that it could happen, that if he continued peeing on himself, there was this option and that it always depended a bit on the child, on how it affected his life. And when we realized that it was really beginning to affect him, we said ‘Well, let’s put a remedy’, we said ‘Well, let’s begin with the pills, and that’s it.’” (I14P16)*

### 3.3 Treatment

#### 3.3.1 Effectiveness and treatment alternatives

Some caregivers and children questioned the efficacy of the medication prescribed. This could be related to past personal or third-party experiences and preferences. In other cases, doubts about adequacy or efficacy arose when there was a delay in diagnosis or instauration of treatment. Finally, some caregivers preferred to exhaust non-pharmacological alternatives before using a medication. In some cases, caregivers wanted to use pharmacological alternatives perceived as more “natural”.

*“And I also knew people who were taking this medicine and it was working well for them, and then I also wanted to take it.” (C5)*

*“And if we could do something different? Medication, relaxation, things that I’ve seen in her that maybe she would need, you know? To take off some pressure from her brothers, the school, everything, you know? And that’s what finally has stopped me from giving her the medication.” (I1P1)*

#### 3.3.2 Side-effects and potency

Caregivers tended to worry about medication side effects. For instance, they might be afraid to initiate a potent medication within the spectrum, as well as medications that are typically prescribed for the adult population.

*“That was what scared us the most, the possible side effects in the sense that we could be fixing a thing and damaging another.” (I11P12)*

*“Knowing that, plus, it was a medication that my mother has taken as an adult for a while. I mean, having a bit the feeling that it’s not a children’s medicine.” (I1F1)*

#### 3.3.3 Duration and dependency

Caregivers were reluctant to initiate a treatment when the expiry date was unclear (e.g., chronic medication), or when a preventive medication, the effects of which were not obtained immediately, was prescribed. This was related to fear of dependency and/or tolerance, which affected willingness to initiate the medication in both caregivers and children.

*“They told us ‘it will be [necessary] for years’ and we said ‘wow, maybe it’s for her entire life, is not it?’” (I11P12)*

“*‒Interviewer: Why do not you like them? Is it because…*

*‒Children: I do not know, because, let’s see, if I take this [pill] one day, and now every day I need [it], then I do not know. Me, I do not like it.” (C2)*

#### 3.3.4 Administration route and cost

The complexity of administration may affect the decision to initiate, especially if it involves discomfort or pain for the child. Although children and caregivers affirmed that the cost of the drug was an influencing factor, caregivers stated that it did not affect the decision if the medication was necessary.

*“They told us that this type of medication, I mean, it implied a prick every month. We were not comfortable at all with that.” (I11P12)*

*“‒Child: They were much more expensive.*

*‒Mother: Yes, yes.*

*‒Child: A cream was more expensive than those pills,*

*‒Mother: Yeah, then*

*‒Child: just one cream.” (I8P9; C5)*

### 3.4 Caregivers’ intrapersonal factors

#### 3.4.1 Emotional burden

Caregivers felt considerable pressure when deciding on their children’s medication, feeling responsible for the harm that giving medication, or withholding it, might cause to their children. This was especially true when the disease or medication was perceived as serious.

*“Your son is everything, right? For example, not giving him a medication that you’ve been prescribed and then having some trouble, jeez! I could not live with it.” (I16P18)*

#### 3.4.2 Health literacy

Caregivers’ health literacy affected their ability to understand healthcare professionals’ recommendations and, therefore, the final decision. Some caregivers and most children adopted a passive role as they believed their health literacy was low.

*“Because you do not have training either, because even if they gave me the information about the medicine, I do not have, I think, enough knowledge to decide the right thing to do, do I?” (I6P6)*

#### 3.4.3 Self-stigma and lack of acceptance

Some caregivers felt embarrassed by their children’s illness and therefore refused to consider treatment. Some even rejected the diagnosis, delaying the initiation of treatment.

*“What has to do with the brain, I mean, at least to me, the perception that I must be very careful with that.” (I12P13)*

*“I felt that it was impossible, that it was not that, that she could not be asthmatic, because I assumed that it was really serious, ok? So, I denied it.” (I17P19)*

### 3.5 Children-related factors

#### 3.5.1 Lack of support

Lack of caregiver support for children regarding their treatment led to non-initiation.

*“‒Children: I say ‘fine, I’ll go [to the pharmacy] later’ and then I had forgotten and that’s it. And they [talking about her educator and tutor] do not remind me either, they do not say anything to me.*

*(…)*

*‒Interviewer: and did your educator or tutor ask about it again, for example? Or not?*

*‒Children: No.” (C2)*

#### 3.5.2 Age

Although participant children were not concerned by age as a factor, it was a concern for caregivers, especially in toddlers.

*“That’s what finally stopped me from giving her the medication, thinking that she was too young.” (I1P1)*

#### 3.5.3 Medicine burden

Caregivers were reluctant to give additional medicines to already polymedicated children.

*“She was a year old, more or less. She was already taking Budesonide every day, morning and afternoon, Montelukast and I do not know what else but, yeah, it was a lot. Then when she got bronchitis, we had to give her Salbutamol. It was, I was overwhelmed by having to give her so much medication” (I11P12)*

#### 3.5.4 Emotional response

Some children reacted emotionally to the diagnosis, especially when perceived as serious, which influenced the caregiver’s decision.

*“No, it did not like me, like, I did not go ‘wow, cool, I’m gonna be medicated’. I was, like, I was beating myself up about it, like, I’m going to be medicated, you know? Something has to help me to be fine. I cannot be fine for myself.” (C6)*

### 3.6 Health professionals

#### 3.6.1 Accessibility

Difficulties accessing clinicians hinder timely diagnosis and treatment and causes uncertainty. In this sense, the relative ease of access to pharmacists may facilitate initiation.

*“I told him [the pharmacist]: ‘Look, I will not buy anything, let’s see if I can get a call from the pediatrician’. And she [the pediatrician] finally called me. It was late because it’s true that day I really needed something already” (I14P16)*

#### 3.6.2 Discourse alignment

Alignment between healthcare professionals encourages caregiver willingness to initiate treatment. Conflicting professional recommendations from the public and private sectors, and between healthcare professionals (e.g., pediatricians and pharmacists), made caregivers insecure and led them to seek second opinions.

*“My pediatrician had already told me that it existed, this type of treatment, of pills, right? A little like a preventive treatment. She had already informed me. And then the neurologist, when he finished all the examination, said, well, he considered that to be the best option.” (I1P1)*

*“Sometimes we have gone to the pharmacy to get this medicine, and they had said ‘But this is not for your eyes, whatever!’ I mean, these things, they make you mistrust the doctor a lot” (C4)*

#### 3.6.3 Information, respect and emotional support

A positive experience with clinician care favored initiation. Clarity, sincerity, and transparency in health professionals generated confidence in caregivers, while insufficient consultation time for queries caused distrust. Respect and emotional support from the prescriber also generated faith in the prescription and facilitated decision-making.

*“That the physician explains all the procedure, how it works, and what it is for exactly. For them to be clear and explain it all properly. If they give me a correct reasoning, I do not need to doubt their word.” (I18P20)*

*“To me, I think it is the most important thing. That throughout the process, whatever the disease or illness may be or whatever, you feel accompanied by the professionals, you know?” (I11P12)*

#### 3.6.4 Professional trust

Professionals’ familiarity with children’s medical history and follow-up promoted trust. While some caregivers who passively accepted and trusted healthcare professionals’ recommendations, others were more circumspect. Some caregivers thought pharmacists’ recommendations were motivated by commercial interests.

*“Well, if you are attended by a physician who has never seen your kid, of course, no matter how much you explain” (I18P18)*

*“Several times, I mean, I have gone to see the doctor, and my mum, she has doubted, or well, even I have doubted because sometimes, moreover, he did not seem sure” (P4)*

#### 3.6.5 Specialty

Not knowing whether the clinician was a pediatrician or specialist generated distrust in the medication and dosage, especially in the emergency department.

*“[You] do not know if they are pediatricians or not. I think that sometimes dosing is also different for a kid or an adult. And you also worry and say ‘Is he right? It is a kid, right?’ Well, you do not know if he’s a pediatrician” (I16P18)*3.7

### 3.7 Context

#### 3.7.1 Media

When in doubt about medication, caregivers often first consulted the doctor or sought information before deciding. Alarming information in the media and on the internet influenced caregivers, who felt insecure about the validity of online information, opting to rely solely on information provided by doctors. Although children rarely searched the internet, they reported the information they found to be reassuring rather than worrisome or alarming.

*“No, I did not searched for information, I did not. Usually, I never look for information on the internet because I want to avoid, who knows what can come up, you know?” (I14P16)*

#### 3.7.2 Peer pressure

The influence exerted by a social group (e.g., the family) to bring about a change in certain attitudes, thoughts, or even values is reflected in the pressure felt by some caregivers.

*“Comments such as ‘No, the kid does not need this’. In my family, for example, I’ve been told, ‘How could you give him medication for his head?’” (I12P14)*

#### 3.7.3 Social stigma

Caregivers often recognized the social stigma associated with their child’s medication or illness, such as negative thoughts or beliefs in the school environment.

*“Since it’s something [the medication] that he takes home, no. For him, it’s worse to pee himself or going on a trip and be seen, for their classmates to see him with a diaper, than taking the medication” (I14P16)*

### 3.8 Healthcare system

#### 3.8.1 Trust in healthcare system

In general, caregivers trust the public health system, although others note a lack of coordination between specialist teams at the national level, or pharmaceutical industry agreements influencing prescribed treatments, which deter them from initiating.

*“We do realize that there are agreements with labs, even the public health system, that benefit them by prescribing one lab or another.” (I5P5)*

#### 3.8.2 Telematics consultation

Caregivers felt that attention received during telephone consultations was inadequate, and this affected their decision to initiate treatment.

*“But the visit was on the phone (…) When I got the message, I said ‘What I want is for my daughter to be visited, not just being called and being visited over the phone’” (I5P5)*

## 4 Discussion

This qualitative study explored, for the first time, pediatric medication initiation for a wide range of illnesses and treatments from the perspective of caregivers and children. The decision to initiate treatment was mainly made by mothers (Ge et al., 2022), preferably in agreement with healthcare professionals, with children only rarely involved. Caregivers evaluated benefit/risk balance based on their perception of the disease and medication, influenced by caregiver/child factors (e.g., stigma, emotional reactions, and health literacy) and healthcare professionals/system factors (e.g., trust, quality of care, information, and accessibility). Context also affected illness and medication perception.

In line with a previous study on tuberculosis, disease severity and the absence of symptoms influenced caregivers’ decisions regarding diagnosis and treatment, limiting children’s access to care even with a confirmed diagnosis (Coit et al., 2020). Caregivers felt intense pressure to make decisions about their children’s health, fearing both side effects and the consequences of not receiving treatment.

As with studies on the initiation of antiretroviral treatment and attention-deficit/hyperactivity disorder (Ahmed et al., 2017; Coletti et al., 2012), mothers’ emotional reactions to diagnosis and treatment recommendation -affected by the children’s emotional responses, self-stigma, and social stigma (Lazaratou et al., 2007)- further impacted decision-making. Failure to accept a diagnosis ranges from 19% to 64% (Lord et al., 2008; Milshtein et al., 2010; Barak-Levy and Atzaba-Poria, 2013; Oppenheim et al., 2024; Baiocco et al., 2017) and is linked to higher maternal distress (Lord et al., 2008; Kearney et al., 2011; Krstić et al., 2015), parental depression (Kearney et al., 2011; Krstić et al., 2015), poorer emotional support (Sheeran et al., 1997), more avoidance strategies (Freda et al., 2015), and lower maternal sensitivity (Oppenheim et al., 2024). Healthcare professionals should assist caregivers in coping with both the diagnosis and the recommended treatment.

Previous studies on adherence described a triadic partnership involving three interactions: children-medical team, child-caregiver and medical team-caregiver (De Civita and Dobkin, 2004). However, we did not identify child-physician interactions when deciding on treatment initiation, even when it was actively explored. This supports our hypothesis that differences between initiation and other forms of (non-)adherence (Vrijens et al., 2012) require further exploration. It may also reflect a paternalistic and hierarchical model that overlooks children’s rights to participate in medical decisions. While caregiver involvement is essential, mature children can take on greater responsibility for their treatment plans. Respecting their views is a fundamental right of child (OHCHR, 2024). Clinicians should assess whether families have accurate medication information, and both caregivers and healthcare professionals should tailor the information to children, considering their preferences (Jordan et al., 2020; Adams et al., 2017; Kon and Morrison, 2018). Evidence on effectiveness and potential side effects should be intelligible to caregivers and children. Non-pharmacological alternatives should also be presented, and stigma must be addressed to facilitate informed decision-making (Adams et al., 2017; Kon and Morrison, 2018).

The discourse of interviewed children aligned with that of caregivers, with one exception: according to children, treatment costs may influence the decision to initiate treatment, something caregivers denied. Quantitative studies show that treatment costs and caregiver socioeconomic status are among the most influential factors in treatment initiation in children (Carbonell-Duacastella et al., 2022). Previous studies exploring factors affecting initiation in adults identified costs as a relevant factor when informants were healthcare professionals, while patients rarely mentioned it (Gil-Girbau et al., 2020; Peñarrubia-María et al., 2022). Although co-payment schemes in Spain consider income in determining the co-payment ratio, populations with lower socioeconomic status are disproportionately burdened (Aznar-Lou et al., 2018). Furthermore, although generally applied to low-intrinsic-value treatments, some medications are excluded from public financing, resulting in 100% co-payment. In our context, this disproportionately affects children with severe diseases (Rubio-Valera et al., 2021).

### 4.1 Limitations

Some limitations should be considered. The initial sampling strategy aimed to include caregivers of children -and the children themselves- who had not started a newly prescribed treatment. However, due to recruitment challenges, we ultimately included caregivers of children who had received a first prescription, regardless of initiation status. Although we believe that the factors influencing decision-making may be similar regardless of whether treatment was initiated, we cannot confirm this. Despite employing an intensive, purposeful search strategy to identify participants, finding informants from specific groups, such as foreign nationals, proved difficult. Health literacy was inferred from the discourse analysis, and only the highest level of education attained was collected in the sociodemographic questionnaire. The study may have been affected by desirability bias. During interviews, feelings of being morally judged were detected in both caregivers and children, potentially masking true thoughts, especially in cases of non-initiation. Some interviews may also have been affected by recall bias. Finally, the COVID-19 pandemic affected both recruitment and data collection methods, which could have influenced the results. However, participants were young and comfortable with online platforms used to conduct the interviews.

## 5 Conclusion

The decision to initiate medication in the pediatric population is multifactorial and influenced by perceptions about the disease and treatment, intrapersonal factors related to both caregivers and children, interpersonal relationships, and healthcare system and contextual factors. Mothers should be the primary target group for interventions aimed at improving medication initiation in children. Healthcare professionals should promote informed decision-making by acknowledging caregivers’ emotional burdens and addressing external influences such as stigma. While mothers are the primary decision-makers in pediatric medication initiation, it is imperative to make greater efforts to include children in decisions concerning their health. Children appear to be largely excluded from this process, and they should receive support from both healthcare professionals and caregivers to help them cope with treatment. Potential strategies include using age-appropriate communication techniques, such as child-friendly educational materials, which could foster a sense of participation and control, even in younger children. Future interventions could explore the development of family-centered decision-making models that involved both caregivers and children, where appropriate.

## Data Availability

The datasets presented in this article are not readily available because Data would allow desanonimization. The ethics committee prevented us from sharing it. No requests to access the datasets will be accepted.
