# Supplementary material for: Insights on pediatric medication initiation: perceptions of caregivers and children
Source: Front Pharmacol. 2025 Jun 6;16:1612169. doi: 10.3389/fphar.2025.1612169 (PMC12179087; doi:10.3389/fphar.2025.1612169)
Supplement: Supplementary file 1 [file Supplementaryfile1.docx]

Supplementary File

**I. Semi-structured thematic guide**

| Beliefs about the illness | Causes, consequences, identity, treatments.  Parent/caregiver perception of the illness.  Children perception of the illness.  Previous experiences with the illness. |
| --- | --- |
| Beliefs about treatment | Benefits and risks.  Type of treatment and characteristics.  Previous experiences.  Alternatives to the prescribed treatment. |
| Relationship with the prescriber | Motives for visiting and expectations.  Healthcare professional – parent and children (family) interaction.  Information received and doubts. |
| Social context | Social norms.  Influence of other healthcare professionals.  Influence of other people (e.g. peers).  Media and other sources of information. |
| COVID-19 | Influence of the COVID-19. |
| Diagnosis* | Diagnostic procedure. |

*Added to the thematic guide after the initial interviews.

**II. Table S1. Research team characteristics (in January 2022)**

| Name | Gender | Age | Training | Main occupation | Main work setting | Experience in qualitative research (years) | Mother |
| --- | --- | --- | --- | --- | --- | --- | --- |
| Maria Rubio-Valera | Fem. | 38 | Pharmacist; PhD | Quality improvement | Secondary care centre | 11 | Yes |
| Cristina Carbonell- Duacastella | Fem. | 30 | Pharmacist | Research | Research unit | 0 | No |
| Montserrat Gil-Girbau | Fem. | 56 | Pharmacist; PhD | Research | Research unit | 7 | Yes |
| Patricia Gabriela-Ricciardelli | Fem. | 62 | Pediatrician | Pediatrician | Secondary care centre | 0 | Yes |
| Eva Pacheco | Fem. | 49 | Pediatrician | Pediatrician | Primary care centre | 0 | Yes |
| Maite Peñarrubia-María | Fem. | 48 | Family physician | Research | Primary care centre | 4 | Yes |
| Ignacio Aznar-Lou | Male | 30 | Pharmacist; PhD | Research | Research unit | 0 | No |

Fem.: Female.
